# Coronaviruses: a challenge of today and a call for extended human postmortem brain analyses

**DOI:** 10.1007/s00702-020-02230-x

**Published:** 2020-07-28

**Authors:** Peter Riederer, Volker ter Meulen

**Affiliations:** 1 Clinic and Policlinic for Psychiatry, Psychosomatics and Psychotherapy, University Hospital Würzburg, Margarete-Hoeppel-Platz 1, 97080 Würzburg, Germany; 2 University of Southern Denmark Odense, J.B. Winslows Vey 18, 5000 Odense, Denmark; 3 Institut für Virologie und Immunbiologie, Universität Würzburg, Versbacherstraße Straße 7, 97078 Würzburg, Germany

**Keywords:** Coronavirus, COVID-19, SARS-CoV-2 brain disorders, Cardiorespiratory centre, Brain pathology, Neurological symptoms/disorders, Brain stem, Parkinson’s disease, Parkinsonism, Alzheimer’s disease, Multiple sclerosis, Movement disorders, Neuroinvasion, Therapy, Neuroprotection, Depression, Cognitive dysfunction, Brain bank, Postmortem studies

## Abstract

While there is abounding literature on virus-induced pathology in general and coronavirus in particular, recent evidence accumulates showing distinct and deleterious brain affection. As the respiratory tract connects to the brain without protection of the blood–brain barrier, SARS-CoV-2 might in the early invasive phase attack the cardiorespiratory centres located in the medulla/pons areas, giving rise to disturbances of respiration and cardiac problems. Furthermore, brainstem regions are at risk to lose their functional integrity. Therefore, long-term neurological as well as psychiatric symptomatology and eventual respective disorders cannot be excluded as evidenced from influenza-A triggered post-encephalitic Parkinsonism and HIV-1 triggered AIDS–dementia complex. From the available evidences for coronavirus-induced brain pathology, this review concludes a number of unmet needs for further research strategies like human postmortem brain analyses. SARS-CoV-2 mirroring experimental animal brain studies, characterization of time-dependent and region-dependent spreading behaviours of coronaviruses, enlightening of pathological mechanisms after coronavirus infection using long-term animal models and clinical observations of patients having had COVID-19 infection are calling to develop both protective strategies and drug discoveries to avoid early and late coronavirus-induced functional brain disturbances, symptoms and eventually disorders. To fight SARS-CoV-2, it is an urgent need to enforce clinical, molecular biological, neurochemical and genetic research including brain-related studies on a worldwide harmonized basis.

## Introduction

Ever since the landmark observations of Constantin von Economo and Rene Cruchet in 1917 and subsequent publications on encephalitis lethargica, viral infections of the central nervous system (CNS) have been of great interest to neurology and neurovirology to study diseases with long-term neurological and psychiatric symptoms of unknown aetiology. Great progress has been made ever since and new CNS diseases such as subacute sclerosing panencephalitis (SSPE) or progressive multifocal leucoencephalopathy (PML) have been linked to specific virus infections. Also the group of coronaviruses which are widespread in nature, infecting animal and men and causing a variety of acute, subacute and chronic diseases, have been studied with respect to CNS involvement. It is, therefore, not surprising that in the corona virus disease 2019 (COVID-19) pandemic, CNS involvement was noted.

In the current COVID-19 pandemic, the respiratory tract is a major target of infection but some reports are showing also clinical involvement of the CNS. It is, therefore, important that not only clinical data of neurological deficits are collected but also studies are carried out to look for acute or late CNS changes with or without virus presence.

### Early pathology of SARS-CoV-2 infection

Fever, cough, sore throat and dyspnea are early and rather unspecific symptoms of coronavirus infections and even before its molecular detection. Pharyngodynia, nasal congestion, rhinorrhoea, smell and taste dysfunctions have been recently described as major symptoms of severe acute respiratory syndrome coronavirus 2 (SARS-CoV-2) (Lovato and de Felippis 2020; Krajewska et al. 2020; Vetter et al. 2020; Yan et al. 2020; Table 1).

**Table 1:** Early symptoms of SARS-CoV-2 infection

Symptomology	In % of patients	References
Fever	85	Lovato and de Filippis (2020)
Cough	68.7	Lovato and de Filippis (2020), Krajewska et al. (2020)
Sore throat		Krajewska et al. (2020)
Dyspnea		Krajewska et al. (2020)
Pharyngodynya	12.4	Lovato and de Filippis (2020), Krajewska et al. (2020)
Nasal congestion	3.7	Lovato and de Filippis (2020), Krajewska et al. (2020)
Rhinorrhea		Krajewska et al. (2020)
Smell dysfunction	68; up to 53; 98; 85,6	Yan et al. (2020), Vetter et al. (2020), Moein et al. (2020), Lechien et al. (2020)
Anosmia (58% of *n* = 60)	25	Moein et al. (2020)
Microsmia	33	Moein et al. (2020)
Moderate microsmia	27	Moein et al. (2020)
Mild microsmia	13	Moein et al. (2020)
Normosmia	2	Moein et al. (2020)
Smell and taste dysfunction	39.2	Beltran-Corbellini et al. (2020), Xydakis et al. (2020), Roe (2020)
Loss of taste	71	Yan et al. (2020)
Gustative disorders	88	Lechien et al. (2020)
Gastrointestinal symptoms	2–40	Vetter et al. (2020)
Overall rate of conjunctivitis	1.1	Loffredo et al. (2020)
Conjunctivitis in severe cases	3	Loffredo et al. (2020)
Conjunctivitis in non-severe cases	0.7	Loffredo et al. (2020)
Conjunctival symptoms	Up to 32	Wu et al. (2020)
Fatigue	39.4	Lovato and de Filippis (2020)
Dizziness	2–40	Vetter et al. (2020)
Comorbidities		
Hypertension	17–58	Lovato and de Filippis (2020), Li et al. (2020a, b), Zheng et al. (2020), Wang et al. (2020)
Diabetes	3.8; 9.7	Lovato and de Filippis (2020), Li et al. (2020a, b)
Coronary heart disease	3–25	Lovato and de Filippis (2020), Zheng et al. (2020), Wang et al. (2020)
Cardiac injury	8–12	Bansal (2020) Wang et al. (2020)
Arrhythmias	44	Zheng et al. (2020), Wang et al. (2020)
Mortality data		
Ischaemic stroke	74	Varatharaj et al. (2020)
Hypertension	35	Zheng et al. (2020)
Coronary heart disease	17	Zheng et al. (2020)
Venous thromboembolism	58	Wichmann et al. (2020)

“Percentage of patients” is mentioned in only a few publications, while there are a number of reports mentioning symptoms appearing in SARS-CoV-2 patients

Elderly patients and especially those with comorbidities, including obesity, type 2 diabetes, hypertension and coronary heart disease, are at risk for increased severity of COVID-19 pathology and mortality (Butler and Barrientos 2020; Naughton et al. 2020). Indeed, when comparing patients with non-severe infection to those with severe infection, the latter were older and had more such underlying disorders. In addition, patients with more severe infections could show neurological manifestations, such as acute cerebrovascular diseases, impaired consciousness and skeletal muscle injury (Mao et al. 2020; Varatharaj et al. 2020).

Only a few publications and sometimes only case reports report on autopsy studies of patients dying from COVID-19 infection (Solomon et al. 2020). Weyhern et al. (2020) report the findings of six autopsies. Besides viral pneumonia, a pronounced CNS involvement with pan-encephalitis, meningitis and brain stem neuronal cell damage was a key event in all cases. CNS haemorrhage was fatal in patients younger than the age of 65 (Weyhern et al. 2020). In 125 patients with complete datasets, 62% presented with a cerebrovascular event, including ischaemic stroke, intracerebral haemorrhage and one CNS vasculitis. 31% presented with altered mental status, including unspecific encephalopathy, encephalitis, neuropsychiatric disorders, psychosis, dementia-like neurocognitive syndrome and affective disorders (Varatharaj et al. 2020). Encephalopathies, inflammatory CNS syndromes, ischaemic strokes and peripheral neurological disorders have been reported by Paterson et al. (2020). Autopsy studies of 18 consecutive patients with SARS-CoV-2 infection who died within 32 days after the onset of symptoms showed only hypoxic changes and did not show encephalitis. In a case report, an autopsy by Reichard et al. (2020) revealed a range of neuropathological lesions with features resembling both vascular and demyelinating etiologies.

Neurological disorders may be caused by COVID-19 due to direct infection of the brain and/or via strong activation of the immune system (Rossmann 2020; Butowt and Bilinska 2020) (Table 2).

**Table 2:** Early neurological/psychiatric symptoms of patients with coronavirus/SARS-CoV-2

Symptomology	In % of patients	References
Neurological symptoms	36.4–84	Mao et al. (2020), Rossmann (2020), Vetter et al. (2020), Bohmwald et al. (2018), Arbour et al. (2000), Burks et al. (1980), Hung et al. (2003), Lau et al. (2004), Yeh et al. (2004), Li et al. (2020a, b), Poyiadji et al. (2020), Roe (2020), Helms et al. (2020)
Encephalopathies/encephalitis	18–23	Varatharaj et al. (2020), Paterson et al. (2020), Weyhern et al. (2020)
Impaired consciousness	15–34	Mao et al. (2020), Rossmann (2020), Rogers et al. (2020), Vetter et al. (2020), Bohmwald et al. (2018), Arbour et al. (2000), Burks et al. (1980), Hung et al. (2003), Lau et al. (2004), Yeh et al. (2004), Li et al. (2020a, b), Poyiadji et al. (2020), Saad et al. (2014), Pinzon et al. (2020), Varatharaj et al. (2020)
Confusion	18–65	Saad et al. (2014), Helms et al. (2020), Rogers et al. (2020)
Cerebrovascular diseases	5.7–8.5	Mao et al. (2020), Rossmann (2020), Vetter et al. (2020), Bohmwald et al. (2018), Arbour et al. (2000), Burks et al. (1980), Hung et al. (2003), Lau et al. (2004), Yeh et al. (2004), Li et al. (2020a, b), Poyiadji et al. (2020), Pinzon et al. (2020), Varatharaj et al. (2020)
Stroke	Up to 74	Hess et al. (2020), Varatharaj et al. (2020), Paterson et al. (2020)
Skeletal muscle injury	19.3	Mao et al. (2020), Rossmann (2020), Vetter et al. (2020), Bohmwald et al. (2018), Arbour et al. (2000), Burks et al. (1980), Hung et al. (2003), Lau et al. (2004), Yeh et al. (2004), Li et al. (2020a, b), Poyladji et al. (2020), Schoser et al (2020)
Myalgia	13.4–71	Pinzon et al. (2020), Saad et al. (2014)
Guillain–Barré syndrome		Vetter et al. (2020), Paterson et al. (2020)
Seizures	8.6	Saad et al. (2014)
Altered mental state	31.0	Varatharaj et al. (2020)
Acute illness		
Depression	32.6	Rogers et al. (2020)
Anxiety	35.7	Rogers et al. (2020)
Insomnia	41.9	Rogers et al. (2020)
Psychosis	0.7	Rogers et al. (2020)
Post-illness		
PTSD (Posttraumatic stress disorder)	32.2	Rogers et al. (2020)
Depression	14.9	Rogers et al. (2020)
Anxiety disorders	14.8	Rogers et al. (2020)
Return to work at follow-up time of 35.3 month	76.9	Rogers et al. (2020)
Agitation	69	Rogers et al. (2020)

“Percentage of patients” is mentioned in only a few publications, while there are a number of reports mentioning symptoms appearing in SARS-CoV-2 patients

Viruses seem to enter the brain via distinct routes either by haematogenous dissemination or neuronal retrograde transport (Desforges et al. 2014; Vetter et al. 2020; Bohmwald et al. 2018). It is assumed that SARS-CoV-2 spreads from peripheral organs, like the gastrointestinal tract, the lung, nose, other organs to the brain. Enhanced binding of SARS-CoV-2 to the nasal cavity olfactory epithelium has been suggested as primary target, as the olfactory epithelium lining blood vessels express two host receptors, ACE-2 and TMPRSS2 proteases, which facilitate virus binding, replication and accumulation (Butowt and Bilinska 2020; Sungnak et al. 2020). Indeed, the infection of the olfactorial epithelium seems to be responsible for olfactory dysfunction and loss of smell in patients with COVID-19 (Butowt and Bilinska 2020; Sungnak et al. 2020). ACE-2 may be related to both respiratory and myocardial injury, because ACE-2 is widely expressed like in the lungs and the cardiovascular system as pointed out by several authors. It is regarded that ACE-2 is a potential risk factor for both respiratory and cardiac failures in patients with COVID-19 (Long et al 2020; Zheng et al. 2020; Cure and Cumhur Cure 2020; Hess et al. 2020; Yang et al. 2020; Bonow et al. 2020; Driggin et al. 2020; Wang et al. 2020).

Spreading from the respiratory tract to the brain is suggested to be based on (1) virus transport to pass from the respiratory tract to the blood and then across the blood–brain barrier into the brain (Rossman 2020; Butowt and Bilinska 2020; Bohmwald et al. 2018) by an transendothelial mechanism by infection of endothelial cells or via destabilisation of tight junctions by inflammatory processes, as well as (2) via infection of olfactory receptor neurons, (3) via diffusion through channels formed by olfactory ensheathing cells (van Riel et al. 2015; Bohmwald et al. 2018) and (4) inflammatory processes affecting the vagus nerve (Amor et al. 2010). Transsynaptic transport and microfusion may occur and cause damage from infection of nerve cells per se and/or immune response (van Riel et al. 2015).

Of special interest is that axons projecting from the olfactory system to the brain lack protection by the blood–brain barrier (BBB) (Broadwell and Jacobowitz 1976) which allows spreading of the virus from the olfactory system to the CNS in rather short time. Experimental studies in mice with HCoV-OC43 infection showed, that neuroinvasion could be demonstrated in the olfactory bulb area after 3 days. Already at 7 days post-infection neuroinvasion of the hippocampus was evident and motor symptoms developed with progressive severity until death of the infected mice at about 20 days post-infection (Jacomy and Talbot 2003; Jacomy et al. 2006).

Of interest are multiexperiment matrix (MEM) data showing a coexpression link of ACE-2 and aromatic aminoacid-decarboxylase (DDC), the enzyme responsible for the synthesis of dopamine and finally noradrenaline and adrenaline from L-DOPA and serotonin from 5-hydroxytryptophan. As SARS-CoV including SARS-CoV-2 down-regulates ACE-2, this might change both the activities of the catecholamine as well as the serotonin pathways (Nataf 2020). ACE-2 knockout mice generated substantially low levels of serotonin (Klempin et al. 2018). These data demonstrate that involvement of neurotransmitter action and pathology is of importance and should be considered in more detail.

Smell is significantly affected in SARS-CoV-2 infected patients which may be the result of virus induced pathology of olfactory sensory neurons in the olfactory epithelium. The olfactory bulb is an important relay as it transforms signals from the olfactory sensory neurons to other parts of the olfactory system including the anterior olfactory nucleus, the olfactory tubercle, amygdala, piriform cortex and entorhinal cortex. Neurotransmitters, as acetylcholine and biogenic amines are involved in transmitting odorant perception (Rothermel et al. 2014; Kapoor et al. 2016; Shea et al. 2008). Taste dysfunctions have been reported in patients suffering from COVID-19 infection (Xydakis et al. 2020; Spinato et al. 2020; Bousquet et al. 2020; Beltran-Corbellini et al. 2020; Gautier and Ravussin 2020; Cecarelli et al. 2020; Lechien et al. 2020; Moein et al. 2020; Sungnak et al. 2020) (Table 1). It has been suggested, that virus attacks the cranial nerves related to smell or the muscosal tissue surrounding these nerves. Inflammatory processes induced by viral infection are of major pathological interest in this regard (Huber 2020).

With regard to coronavirus infection and especially SARS-CoV-2 of the brain, it may be of particular interest to consider infection of both the brain respiratory centre located in the medulla–pons areas and the medullary cardiovascular centres. Viral infection of those centres, which are responsible for generating and maintaining rhythms of respiration and cardiovascular activity, may disturb and even disrupt the underlying homeostasis with environmental stimuli. Disturbance or even disruptions of those pathways are risk factors which may lead and contribute to the severeness of the disease process of COVID-19 patients (Gandhi et al. 2020; Lucchese and Flöel 2020). Cardiac dysfunctions have been observed in patients with severe viral infection and patients with comorbidities like respiratory disorders, diabetes type II, obesity, hypertension, coronary heart disease, myocardial injury, myocarditis, acute myocardial infarction, heart failure, dysrhythmias and venous thromboembolic events (Li et al. 2020b; Bansal 2020; Long et al. 2020; Zheng et al. 2020) (Table 1) do have additional risk factors for the outcome of the disease process.

Recent clinical evidence shows that SARS-CoV-2 induces neuromuscular symptoms (Schoser et al. 2020) with muscle pain and weakness and fatigue. In these patients, 11% revealed an increase of creatinkinase presenting with muscle weakness. Creatinkinase increased significantly with clinical severity from 22.4% in non-complicated patients to 71% of critically ill patients (Schoser et al. 2020) (Table 2).

### Brain pathology of coronavirus infection in experiments in animals

The neurotropism of certain animal coronaviruses has lead in the past to experimental studies in mice and rats to investigate the conditions leading to CNS damage using the mouse hepatitis coronavirus strain JHM (Nagashima et al. 1978a, b).

Three types of diseases were observed: (1) acute panencephalitis with demyelinating foci and affection of oligodendroglial cells and neurons, (2) subacute demyelinating encephalomyelitis three weeks after virus infection of new-born and weanling rats, which in about 35% developed paralysis. Demyelination was observed predominantly in the white matter of brainstem, pons, optic nerve and spinal cord. Axons and neurons were well preserved whereas virus was only detectable in oligodendroglial cells, (3) chronic progressive paralysis in 5% of infected animals 6–8 months later (Weiner 1973; Nagashima et al. 1978a, b). Of interest is the notion, that remyelination for both, peripheral and central nervous system could be observed in clinically silent animals (Nagashima et al. 1979). In both infected mice and rats, infectious virus could be isolated from brain tissue during the acute or subacute stage of encephalitis. Thereafter, infectious virus disappeared but viral antigen persisted (Sörensen and Dales 1985).

More recent animal studies using mice transgenic for the SARS-CoV receptor ACE-2 demonstrated that viral brain infection covered all brain regions time dependently and complete after 4 days. Neurons were highly susceptible for SARS-CoV and prevention of severe murine disease could be reached only by absence of the host cell receptor (Netland et al. 2008). As neither apoptosis or necrosis, nor inflammation could be verified in these studies, the authors speculated, that non-inflammatory processes like autophagy may be involved in the neuronal loss in SARS-CoV-infected K18-h ACE-2 mice without encephalitis (Netland et al. 2008). On the basis of findings in animal experiments, the question arises if COVID-19 patients with CNS infection could develop later CNS degenerative disorders as a consequence of an infection of specific cell populations.

## Implications for neurodegenerative disorders

Animal studies point to the view that glial cells and oligodendroglial cells are of particular vulnerability for coronavirus infection (Barach-Latas et al. 1997). Acute and persistent infections of neural cell lines with human coronavirus OC43 and 229E confirm sensitivity of glial cells towards virus infection (Bonavia et al 1997; Arbour et al 1999a, b). Infection of human astrocytic cell line U-373MG by the OC43 strain of human coronavirus resulted in an increase of IL-6, TNF-ɑ and MCP-1 mRNA expression and modulation of the activity of matrix metalloproteinase-2 and -9. Nitric oxide production was notable in U-373MG cells as well as in microglial cell line CHME-5, indicating that coronavirus may contribute to the pathogenesis of multiple sclerosis (Edwards et al 2000). Infection by HCoV-OC43 with a single-point mutation in the spike protein led to a hind-limb paralytic disease in infected mice (Brison et al. 2011). This infection resulted in glutamatergic excitotoxicity, which could be antagonized by an inhibitor of AMPA receptors, GYKI-52466, which was accompanied by improvement of clinical scores and protection of CNS from neuronal dysfunction (Brison et al. 2011).

In three patients suffering from MERS-CoV T2-weighted MRI imaging showed striking changes characterized by widespread, bilateral hyperintense lesions within the white matter and subcortical areas of the frontal, temporal and parietal lobes, the basal ganglia and corpus callosum, giving raise to the possibility, that MERS-CoV may lead to long-lasting severe alterations of brain tissue (Arabi et al. 2015). As described in more detail by Matias-Guiu et al. (2020), coronavirus-like particles have been identified in autopsied brain tissue (Burks et al. 1980; Murray et al. 1992; Stewart et al. 1992; Dessau et al. 2001), as well as detection of antibodies to human coronavirus (Salmi et al. 1982) and CoV RNA in the CSF of patients with multiple sclerosis (Cristallo et al. 1997).

### Parkinsonism

Infectious agents associated with Parkinsonism are influenza A, HIV, measles, Japanese B encephalitis, Western equine encephalitis, tick-borne encephalitis, polyomyelitis and cytomegalovirus (Nisipeanu et al. 1997). Neuromelanin of the substantia nigra pars compacta is of special interest in this regard as it (1) is an immune stimulator (Oberländer et al. 2011) and (2) pigmented neurons of the SN were significantly decreased in HIV-1-infected brains (Itoh et al. 2000), thus contributing to dopaminergic pathology. A viral hypothesis for Parkinson’s disease has been suggested for long time, namely since the influenza A pandemia 1915–1927 with post-encephalitic Parkinsonism as fatal consequence years later (Foley 2009; Lutters et al. 2018; Elizan and Casal 1983; Takahashi and Yamada 2001; Hawkes et al. 2007). Any specific viral antibodies, RNA, viral particles or inclusions could not be detected in several studies in brain tissue (Martilla et al. 1977; Elizan et al. 1979; Jellinger 2001; McCall et al. 2001; Schwartz and Elizan 1979; Gamboa et al. 1974). These data contrast to those reported by Mihara et al. (2001) and Rohn and Catlin (2011), who have detected immunolocalization of influenza A virus in PD brain (Rohn and Catlin 2011) and isolated lesions in the bilateral SN on MRI associated with influenza A (Mihara et al 2001). As summarized by Oliver et al. (1997a), changes in the dopaminergic system have been observed in many studies following virus infection and encephalitis. Indeed, dopaminergic neurons are heavily involved in the HIV-1 pathology using SIVmac 251 infection in rhesus monkeys as well as in a retroviral rat model of HIV and murine leucemia virus (MULV)NT 40 infections (Koutsilieri et al. 2001a, b, 2002a; b; Czub et al. 2001). Experimental long-term studies with H5N1 influenza virus in mice showed that intranasal application of H5N1 induces transient loss of dopamine in the substantia nigra pars compacta (SNpc) and basal ganglia. In addition, activated microglia and increase in cytokines could be detected suggesting, that viral infection may not be excluded as trigger for Parkinsonism (Jang et al. 2012).

From this experimental approach, it is hypothesized that (1) viral infection of the substantia nigra pars compacta is at risk for the development of Parkinsonism and (2), as Parkinson’s disease is common in the elderly and Parkinson’s disease clinically shows compromise of the respiratory and cardiac systems,  Parkinson’s disease is at risk for SARS-CoV-2 infection (Helmich and Bloem 2020). Indeed, HCoV has been detected in brain tissue from Parkinson’s disease (Fazzini et al. 1992; Arbour et al. 2000). Long-term clinical observations of patients with COVID-19 infection will show, whether SARS-CoV-2 triggers Parkinsonism and/or depression in genetically vulnerable human beings.

### Psychiatric symptomology

Arbour et al. (2000) have also detected HCoV-229E- and HCoV-0C43 RT-PCR-positive results in rare cases of amyotrophic lateral sclerosis (ALS), Alzheimer disease, depression and schizophrenia. SARS-CoV long-term adverse reactions, like depression and other psychiatric symptomology have not been reported in great detail. However, there is preliminary evidence to assume that depression, fatigue and sleep disturbances are evident in post-SARS-CoV patients (Moldofsky and Patcai 2011). The most recent publication by Rogers et al. (2020) gives a detailed representation of psychiatric symptoms of SARS, MERS and COVID-19 patients in the acute as well as in the post illness phases (Table 2). Furthermore, it cannot be excluded that in the process of SARS-CoV-2 CNS infections, impaired consciousness occurs, which my lead to cognitive deficiencies.

Indeed, besides cerebrovascular events, altered mental status was the second most common presentation compromising encephalopathy or encephalitis and primary psychiatric diagnosis, often occurring in younger patients (Varatharaj et al. 2020) (Table 2).

### Experimental observations in the protection of brain cell damage

Since we cannot exclude the possibility that SARS-COV-2 could lead to CNS damage, it is worthwhile to discuss experimental data obtained from pharmacological basic research. Enlargement of the therapeutic armamentarium for drugs protecting from virus-induced damage is scarce and limited to human case reports, experimental approaches using disease-related animal models and in vitro studies. To mention a few ones, the following options have been proposed: therapeutic strategies related to neurotransmitter pathology are targeting ACE-2. As there are close interactions between ACE-2 and nicotinic receptors, nicotine exposure due to smoking has been predicted to enhance the risk for COVID-19 neuroinfection (Kabbani and Olds 2020). Therefore, nicotine receptor antagonists may counteract the risk for SARS-CoV-2 viral brain entry and brain pathology. Even of more interest is the data showing a potentiation of SIV replication by drugs used clinically to substitute loss of dopamine in Parkinson’s disease (Scheller et al 2000). The conclusion of this work possibly is of interest for clinical treatment options in HIV-1 infected patients with a parkinsonism/dementia syndrome. While levodopa and inhibitors of monoamine oxidase B (MAO-I) therapy of parkinsonism is obsolete in this regard (Koutsilieri et al 2002a, b, 2004), treatment with the NMDA-receptor channel antagonists amantadine/memantine are advised from these experimental studies (Meisner et al. 2008; Olney et al. 1989).

Aminoadamantanes, amantadine and memantine have been used for long time in the treatment of Parkinson’s disease (amantadine) and Alzheimer disease (memantine). These drugs are primarily glutamate related NMDA-receptor channel antagonists (Kornhuber et al. 1989, 1991) and inhibit glutamatergic excitotoxicity associated with these neurodegenerative disorders. More recent studies support the antiviral potential of aminoadamantanes, including development of novel compounds (Kesel et al. 2013) and treatment of virus replication (Leibowitz and Reneker 1993) including HCoV-OC43 replication by memantine (Brison et al. 2014). Most recently, Hasanagic and Serdarevic (2020) suggested that memantine (besides its NMDA-R channel blocking properties) through its α7-nAChR antagonism may counteract proinflammatory cytokines induced in cell cultures by HCoV-OC43. This is of special interest, because α7-nAChR is localized in lungs and in the CNS. As ACE-2 expression is mediated by stimulation of α7-nAChR nicotine (smoking!) might promote entry of SARS-CoV-2 into the respiratory epithelium.

Serotonin antagonists have been proposed too. Cinanserin (SQ10,643) has been studied in bacterially expressed 3 CL pro SARS-CoV and the related human coronavirus 229E. 5microM of cinanserin inhibited the catalytic activity by 50% (Chen et al. 2005). The antiviral activity of cinanserin could be substantiated in tissue culture assays and confirmed strong inhibition of coroanvirus replication (Chen et al. 2005; Yang et al. 2008).

 Interestingly, a very recent screening of substances effective to inhibit SARS-COV-2 showed that the antidepressant serotonin selective reuptake inhibitor (SSRI) fluoxetine inhibited the virus at a concentration of 0.8 µg/ml (Zimniak et al 2020). These studies demonstrated that cianserin and fluoxetine enter at a structural site of the virus which is important for the replication of SARS-CoV-2 and this is independent from the compounds action on the serotonergic system.

An initial “cytokine storm” induced by viral suppression of pineal melatonin has been suggested to contribute to virus-induced brain pathology (Anderson and Reiter 2020). Pineal melatonin is involved in a variety of intermediary cell processes, including the activation of the tricarboxylic acid cycle, oxidative phosphorylation and ATP production, thus regulating mitochondrial and immune cell phenotype (Anderson and Reiter 2020). Drug development to enhance melatonin concentration and function seems to be a useful target to reduce viral infection potential.

NOS2/NO is associated with regulation of chemokine expression and inflammation. Inhibition of NOS2/NO slows the progression of MHV-induced demyelination (Lane et al. 1999). There is also a role for apoD in the regulation of inflammation and suggests that it protects from HVoV-OC43-induced encephalitis, probably through the phospholipase A2 signalling pathway (Do Carmo et al. 2008). Some more recent developments are those directed to treat the acute respiratory distress syndrome (ARDS) (Dreher et al. 2020), which shows inflammation due to acute hypoxemia and diffuse alveolar injury “following a triggering factor” (Santos Nasciemento et al. 2019). These authors propose the development of fluorophenyl imidazole-derived molecules to treat pathologies, in which inflammation, in particular based on p38 MAPK and NFkB, plays a privotal role (Santos Nasciemento et al. 2019).

These examples point to the view that research on transmitter alterations after virus infection might be suitable (1) to gain knowledge about virus induced neuronal pathology of the CNS and (2) to get new targets for developing neuron protective and restorative drugs. For coronavirus, neurochemical, molecular biological/genetic research enlightening nerval participation of coronavirus toxic affection are largely missing.

## Conclusion

Although there is abounding description of virus-induced pathology of peripheral organs, there is lack of evidence as to the viral staging pathology of brain regions and of neuron as well as of glial affection. This, however, seems to be of importance as evidence is accumulating, that viruses and especially coronaviruses including SARS-CoV-2 infect the brain with great affinity to brain regions. Long-term pathological outcome of coronavirus-induced brain affection facilitating or even triggering brain associated disorders like neurodegenerative disorders have to be considered. Therefore, it is important to learn more about SARS-CoV-2-induced brain affection and its short- as well as its long-term consequences. As such targets for future clinical and brain coronavirus-related research and unmet needs are summarized:

1. Human postmortem brain studies are essential to understand HCoV-induced brain pathologies (Ellul et al. 2020; Glatzel 2020), including neuropathology and regional human postmortem neurotransmitter analyses. Moreover, molecular biological and—genetic studies should give evidence for functional disturbances caused by coronavirus affection. Virus affection of neuromelanin containing substantia nigra and locus coeruleus as well as research on coronavirus damaged oligodendrocytes are of importance to understand the vulnerability potential for neurodegenerative disorders.
2. Regional detection of virus footprints and RNA in postmortem brains, as well as spreading characteristics of coronaviral infection/time dependency/staging in animal studies/models and longitudinal studies are necessary to enlighten details of SARS-CoV-2 affinity to brain regions.
3. Studies of mechanisms to explain the differences of coronaviral variations of neuropathology in mice and rat strains as well as in organoids are of importance, in particular, since the pathological outcome of coronavirus infection in various strains of rodents need an explanation (Dörries et al 1987a, b).
4. Age-dependent variety (young/adult/aged) and gender specificity, regarding severity of SARS-CoV-2 neurovirulence should be studied in animal experiments to understand the great divergence of CoV infection rates.
  In addition, studies to the genetic and immunological background of host are regarded as important in this respect. Not much is known to characterise specificity and selectivity of various viral infection factors for resistance against viral attacks.
5. Drug developments to protect neurons and glia from coronavirus induced pathology are of importance to protect nerve tissue from viral toxicity beside SARS-CoV-2 treatment strategies.

Answers to these questions may contribute to understand why SARS-CoV-2 affects aged and young human beings so differently. Age, reduced immunological defence, comorbidity and underlying genetic vulnerabilities are at risk for the severity of viral attack in general and SARS-CoV-2 in particular. In this respect, extensive clinical, neuropathological and molecular biologic/neurochemical postmortem studies as well as animal and in vitro studies are of utmost importance to uncover the enigma of viral infections and its disastrous pathology.

So far, we have learned that beside the respiratory tract as the main organ being infected in humans, the SARS-CoV-2 virus has the potential to spread and infect other organs as well. It has to be seen, to what longlasting deficits may develop.

## References

[CR501] Amor S, Puentes F, Baker D, van der Valk P (2010). Inflammation in neurodegenerative diseases. Immunology.

[CR1] Anderson G, Reiter RJ (2020). Melatonin: roles in influenza, Covid-19, and other viral infections. Rev Med Virol.

[CR2] Arabi YM, Harthi A, Hussein J, Bouchama A, Johani S, Hajeer AH, Saeed BT, Wahbi A, Saedy A, AlDabbagh T, Okaili R, Sadat M, Balkhy H (2015). Severe neurologic syndrome associated with Middle East respiratory syndrome corona virus (MERS-CoV). Infection.

[CR3] Arbour N, Ekandé S, Côté G, Lachance C, Chagnon F, Tardieu M, Cashman NR, Talbot PJ (1999). Persistent infection of human oligodendrocytic and neuroglial cell lines by human coronavirus 229E. J Virol.

[CR4] Arbour N, Côté G, Lachance C, Tardieu M, Cashman NR, Talbot PJ (1999). Acute and persistent infection of human neural cell lines by human coronavirus OC43. J Virol.

[CR5] Arbour N, Day R, Newcombe J, Talbot PJ (2000). Neuroinvasion by human respiratory coronaviruses. J Virol.

[CR6] Bansal M (2020). Cardiovascular disease and COVID-19. Diabetes Metab Syndr.

[CR7] Barac-Latas V, Suchanek G, Breitschopf H, Stuehler A, Wege H, Lassmann H (1997). Patterns of oligodendrocyte pathology in coronavirus-induced subacute demyelinating encephalomyelitis in the Lewis rat. Glia.

[CR8] Beltrán-Corbellini A, Chico-García JL, Martínez-Poles J, Rodríguez-Jorge F, Natera-Villalba E, Gómez-Corral J, Gómez-López A, Monreal E (2020). Acute-onset smell and taste disorders in the context of Covid-19: a pilot multicenter PCR-based case-control study. Eur J Neurol.

[CR10] Bohmwald K, Gálvez NMS, Ríos M, Kalergis AM (2018). Neurologic alterations due to respiratory virus infections. Front Cell Neurosci.

[CR11] Bonavia A, Arbour N, Yong VW, Talbot PJ (1997). Infection of primary cultures of human neural cells by human coronaviruses 229E and OC43. J Virol.

[CR13] Bonow RO, Fonarow GC, O'Gara PT, Yancy CW (2020). Association of coronavirus disease 2019 (COVID-19) with myocardial injury and mortality. JAMA Cardiol.

[CR14] Bousquet J, Akdis C, Jutel M, Bachert C, Klimek L, Agache I, Ansotegui IJ, Bedbrook A, Bosnic-Anticevich S, Canonica GW, Chivato T, Cruz AA, Czarlewski W, Del Giacco S, Du H, Fonseca JA, Gao Y, Haahtela T, Hoffmann-Sommergruber K, Ivancevich JC, Khaltaev N, Knol EF, Kuna P, Larenas-Linnemann D, Mullol J, Naclerio R, Ohta K, Okamoto Y, O'Mahony L, Onorato GL, Papadopoulos NG, Pfaar O, Samolinski B, Schwarze J, Toppila-Salmi S, Teresa Ventura M, Valiulis A, Yorgancioglu A, Zuberbier T, ARIA-MASK study group (2020). Intranasal corticosteroids in allergic rhinitis in COVID-19 infected patients: An ARIA-EAACI statement. Allergy.

[CR15] Brison E, Jacomy H, Desforges M, Talbot PJ (2011). Glutamate excitotoxicity is involved in the induction of paralysis in mice after infection by a human coronavirus with a single point mutation in its spike protein. J Virol.

[CR16] Brison E, Jacomy H, Desforges M, Talbot PJ (2014). Novel treatment with neuroprotective and antiviral properties against a neuroinvasive human respiratory virus. J Virol.

[CR17] Broadwell RD, Jacobowitz DM (1976). Olfactory relationships of the telencephalon and diencephalon in the rabbit. III. The ipsilateral centrifugal fibers to the olfactory bulbar and retrobulbar formations. J Comp Neurol.

[CR18] Burks JS, DeVald BL, Jankovsky LD, Gerdes JC (1980). Two coronaviruses isolated from central nervous system tissue of two multiple sclerosis patients. Science.

[CR19] Butler MJ, Barrientos RM (2020). The impact of nutrition on COVID-19 susceptibility and long-term consequences. Brain Behav Immun.

[CR20] Butowt R, Bilinska K (2020). SARS-CoV-2: olfaction, brain infection, and the urgent need for clinical samples allowing earlier virus detection. ACS Chem Neurosci.

[CR22] Ceccarelli M, Berretta M, Venanzi Rullo E, Nunnari G, Cacopardo B (2020). Differences and similarities between Severe Acute Respiratory Syndrome (SARS)-CoronaVirus (CoV) and SARS-CoV-2. Would a rose by another name smell as sweet?. Eur Rev Med Pharmacol Sci.

[CR23] Chen L, Gui C, Luo X, Yang Q, Günther S, Scandella E, Drosten C, Bai D, He X, Ludewig B, Chen J, Luo H, Yang Y, Yang Y, Zou J, Thiel V, Chen K, Shen J, Shen X, Jiang H (2005). Cinanserin is an inhibitor of the 3C-like proteinase of severe acute respiratory syndrome coronavirus and strongly reduces virus replication in vitro. J Virol.

[CR25] Cristallo A, Gambaro F, Biamonti G, Ferrante P, Battaglia M, Cereda PM (1997). Human coronavirus polyadenylated rna sequences in cerebrospinal fluid from multiple sclerosis patients. New Microbiol.

[CR26] Cure E, Cumhur CM (2020). Angiotensin-converting enzyme inhibitors and angiotensin receptor blockers may be harmful in patients with diabetes during COVID-19 pandemic. Diabetes Metab Syndr.

[CR27] Czub S, Koutsilieri E, Sopper S, Czub M, Stahl-Hennig C, Müller JG, Pedersen V, Gsell W, Heeney JL, Gerlach M, Gosztonyi G, Riederer P, ter Meulen V (2001). Enhancement of central nervous system pathology in early simian immunodeficiency virus infection by dopaminergic drugs. Acta Neuropathol.

[CR29] Desforges M, Le Coupanec A, Brison É, Meessen-Pinard M, Talbot PJ (2014). Human respiratory coronaviruses : neuroinvasive, neurotropic and potentially neurovirulent pathogens. Virologie (Montrouge).

[CR30] Dessau RB, Lisby G, Frederiksen JL (2001). Coronaviruses in brain tissue from patients with multiple sclerosis. Acta Neuropathol.

[CR31] Do Carmo S, Jacomy H, Talbot PF, Rassart E (2008). Neuroprotective effect of apolipoprotein D against human coronavirus OC43-induced encephalitis in mice. J Neurosci.

[CR33] Dörries R, Watanabe R, Wege H, ter Meulen V (1987). Intrathecal humoral immune response in coronavirus induced encephalomyelitis of Lewis and BN rats. Adv Exp Med Biol.

[CR34] Dörries R, Watanabe R, Wege H, ter Meulen V (1987). Analysis of the intrathecal humoral immune response in Brown Norway (BN) rats, infected with the murine coronavirus JHM. J Neuroimmunol.

[CR35] Dos Santos Nascimento MVP, Mattar Munhoz AC, De Campos Facchin BM, Fratoni E, Rossa TA, Mandolesi Sá M, Campa CC, Ciraolo E, Hirsch E, Dalmarco EM (2019). New pre-clinical evidence of anti-inflammatory effect and safety of a substituted fluorophenyl imidazole. Biomed Pharmacother.

[CR36] Dreher M, Kersten A, Bickenbach J, Balfanz P, Hartmann B, Cornelissen C, Daher A, Stöhr R, Kleines M, Lemmen SW, Brokmann JC, Müller T, Müller-Wieland D, Marx G, Nikolaus M (2020). Charakteristik von 50 hospitalisierten COVID-19-Patienten mit und ohne ARDS (The characteristics of 50 hospitalized COVID-19 patients with and without ARDS). Dtsch Arztebl Int.

[CR37] Driggin E, Madhavan MV, Bikdeli B, Chuich T, Laracy J, Biondi-Zoccai G, Brown TS, Der Nigoghossian C, Zidar DA, Haythe J, Brodie D, Beckman JA, Kirtane AJ, Stone GW, Krumholz HM, Parikh SA (2020). Cardiovascular considerations for patients, health care workers, and health systems during the COVID-19 pandemic. J Am Coll Cardiol.

[CR40] Edwards JA, Denis F, Talbot PJ (2000). Activation of glial cells by human coronavirus OC43. Infect J Neuroimmunol.

[CR42] Elizan TS, Casals J (1983). The viral hypothesis in Parkinsonism. J Neural Transm Suppl.

[CR43] Elizan TS, Madden DL, Noble GR, Herrmann KL, Gardner J, Schwartz J, Smith H, Sever JL, Yahr MD (1979). Viral antibodies in serum and CSF of Parkinsonian patients and controls. Arch Neurol.

[CR44] Ellul M, Varatharaj A, Nicholson TR, Pollak TA, Thomas N, Easton A, Zandi MS, Manji H, Solomon T, Carson A, Turner MR, Kneen R, Galea I, Pett S, Thomas RH, Michael BD, Committee CS (2020). Defining causality in COVID-19 and neurological disorders. J Neurol Neurosurg Psychiatry.

[CR45] Fazzini E, Fleming J, Fahn S (1992). Cerebrospinal fluid antibodies to coronavirus in patients with Parkinson's disease. Mov Disord.

[CR46] Foley PB (2009). Encephalitis lethargica and influenza. I. The role of the influenza virus in the influenza pandemic of 1918/1919. J Neural Transm.

[CR47] Gamboa ET, Wolf A, Yahr MD, Harter DH, Duffy PE, Barden H, Hsu KC (1974). Influenza virus antigen in postencephalitic parkinsonism brain. Detection by immunofluorescence. Arch Neurol.

[CR48] Gandhi S, Srivastava AK, Ray U, Tripathi PP (2020). Is the collapse of the respiratory center in the brain responsible for respiratory breakdown in COVID-19 patients?. ACS Chem Neurosci.

[CR49] Gautier JF, Ravussin Y (2020). A new symptom of COVID-19: loss of taste and smell. Obesity (Silver Spring).

[CR50] Glatzel M (2020). Neuropathology of COVID-19: where are the neuropathologists?. Brain Pathol.

[CR54] Hasanagic S, Serdarevic F (2020). Potential role of memantine in the prevention and treatment of COVID-19: its antagonism of nicotinic acetylcholine receptors (nAChR) and beyond. Eur Respir J.

[CR55] Hawkes CH, Del Tredici K, Braak H (2007). Parkinson's disease: a dual-hit hypothesis. Neuropathol Appl Neurobiol.

[CR56] Helmich RC, Bloem BR (2020). The impact of the COVID-19 pandemic on Parkinson's disease: hidden sorrows and emerging opportunities. J Parkinsons Dis.

[CR57] Helms J, Kremer S, Merdji H, Clere-Jehl R, Schenck M, Kummerlen C, Collange O, Boulay C, Fafi-Kremer S, Ohana M, Anheim M, Meziani F (2020). Neurologic features in severe SARS-CoV-2 infection. N Engl J Med.

[CR58] Hess DC, Eldahshan W, Rutkowski E (2020). COVID-19-related stroke. Transl Stroke Res.

[CR59] Huber J (2020) How viruses like the coronavirus can steal our sense of smell. scopeblog.stanford.edu

[CR60] Hung ECW, Chim SSC, Chan PKS, Tong YK, Ng EKO, Chiu RWK, Leung CB, Sung JJY, Tam JS, Lo YMD (2003). Detection of SARS coronavirus RNA in the cerebrospinal fluid of a patient with severe acute respiratory syndrome. Clin Chem.

[CR61] Itoh K, Mehraein P, Weis S (2000). Neuronal damage of the substantia nigra in HIV-1 infected brains. Acta Neuropathol.

[CR62] Jacomy H, Talbot P (2003). Vacuolating encephalitis in mice infected by human coronavirus OC43. J Virol.

[CR63] Jacomy H, Fragoso G, Almazan G, Mushynski WE, Talbot P (2006). Human coronavirus OC43 infection induces chronic encephalitis leading to disabilities in BALB/C mice. J Virol.

[CR64] Jang H, Boltz D, McClaren J, Pani AK, Smeyne M, Korff A, Webster R, Smeyne RJ (2012). Inflammatory effects of highly pathogenic H5N1 influenza virus infection in the CNS of mice. J Neurosci.

[CR65] Jellinger KA (2001). Influenza RNA not detected in archival brain tissues from acute encephalitis lethargica cases or in postencephalitic Parkinson cases. J Neuropathol Exp Neurol.

[CR68] Kabbani N, Olds JL (2020). Does COVID19 infect the brain? If so, smokers might be at a higher risk. Mol Pharmacol.

[CR500] Kapoor V, Provost AC, Agarwal P, Murthy VN (2016). Activation of raphe nuclei triggers rapid and distinct effects on parallel olfactory bulb output channels. Nat Neurosci.

[CR69] Kesel AJ, WeissHC SA, Day CW, Barnard DL, Detorio MA, Schinazi RF (2013). Antiviral agents derived from 1-adamantyl singlet nitrenes. Antiviral Chem Chemother.

[CR70] Klempin F, Mosienko V, Matthes S, Villela DC, Todiras M, Penninger JM, Bader M, Santos RAS, Alenina N (2018). Depletion of angiotensin-converting enzyme 2 reduces brain serotonin and impairs the running-induced neurogenic response. Cell Mol Life Sci.

[CR72] Kornhuber J, Bormann J, Retz W, Hübers M, Riederer P (1989). Memantine displaces [3H]MK-801 at therapeutic concentrations in postmortem human frontal cortex. Eur J Pharmacol.

[CR73] Kornhuber J, Bormann J, Hübers M, Rusche K, Riederer P (1991). Effects of the 1-amino-adamantanes at the MK-801-binding site of the NMDA-receptor-gated ion channel: a human postmortem brain study. Eur J Pharmacol.

[CR75] Koutsilieri E, ter Meulen V, Riederer P (2001). Neurotransmission in HIV associated dementia: a short review. J Neural Transm.

[CR76] Koutsilieri E, Scheller C, Sopper S, Götz ME, Gerlach M, Meulen V, Riederer P (2001). Selegiline completely restores choline acetyltransferase activity deficits in simian immunodeficiency infection. Eur J Pharmacol.

[CR77] Koutsilieri E, Sopper S, Scheller C, ter Meulen V, Riederer P (2002). Parkinsonism in HIV dementia. J Neural Transm.

[CR78] Koutsilieri E, Sopper S, Scheller C, ter Meulen V, Riederer P (2002). Involvement of dopamine in the progression of AIDS Dementia Complex. J Neural Transm.

[CR79] Koutsilieri E, Scheller C, ter Meulen V, Riederer P (2004). Monoamine oxidase inhibition and CNS immunodeficiency infection. Neurotoxicology.

[CR80] Krajewska J, Krajewski W, Zub K, Zatoński T (2020). COVID-19 in otolaryngologist practice: a review of current knowledge. Eur Arch Otorhinolaryngol.

[CR81] Lane TE, Fox HS, Buchmeier MJ (1999). Inhibition of nitric oxide synthase-2 reduces the severity of mouse hepatitis virus-induced demyelination: implications for NOS2/NO regulation of chemokine expression and inflammation. J Neurovirol.

[CR82] Lau KK, Yu WC, Chu CM, Lau ST, Sheng B, Yuen KY (2004). Possible central nervous system infection by SARS coronavirus. Emerg Infect Dis.

[CR83] Lechien JR, Chiesa-Estomba CM, De Siati DR, Horoi M, Le Bon SD, Rodriguez A, Dequanter D, Blecic S, El Afia F, Distinguin L, Chekkoury-Idrissi Y, Hans S, Delgado IL, Calvo-Henriquez C, Lavigne P, Falanga C, Barillari MR, Cammaroto G, Khalife M, Leich P, Souchay C, Rossi C, Journe F, Hsieh J, Edjlali M, Carlier R, Ris L, Lovato A, De Filippis C, Coppee F, Fakhry N, Ayad T, Saussez S (2020). Olfactory and gustatory dysfunctions as a clinical presentation of mild-to-moderate forms of the coronavirus disease (COVID-19): a multicenter European study. Eur Arch Otorhinolaryngol.

[CR84] Leibowitz JL, Reneker SJ (1993). The effect of amantadine on mouse hepatitis virus replication. Adv Exp Med Biol.

[CR86] Li B, Yang J, Zhao F, Zhi L, Wang X, Liu L, Bi Z, Zhao Y (2020). Prevalence and impact of cardiovascular metabolic diseases on COVID-19 in China. Clin Res Cardiol.

[CR87] Li YC, Bai WZ, Hashikawa T (2020). The neuroinvasive potential of SARS-CoV2 may play a role in the respiratory failure of COVID-19 patients. J Med Virol.

[CR89] Loffredo L, Pacella F, Pacella E, Tiscione G, Oliva A, Violi F (2020). Conjunctivitis and COVID-19: a meta-analysis. J Med Virol.

[CR90] Long B, Brady WJ, Koyfman A, Gottlieb M (2020). Cardiovascular complications in COVID-19. Am J Emerg Med.

[CR91] Lovato A, de Filippis C (2020). Clinical presentation of COVID-19: a systematic review focusing on upper airway symptoms. Ear Nose Throat J.

[CR92] Lucchese G, Flöel A (2020). Molecular mimicry between SARS-CoV-2 and respiratory pacemaker neurons. Autoimmun Rev.

[CR93] Lutters B, Foley P, Koehler PJ (2018). The centennial lesson of encephalitis lethargica. Neurology.

[CR94] Mao L, Jin H, Wang M, Hu Y, Chen S, He Q, Chang J, Hong C, Zhou Y, Wang D, Miao X, Li Y, Hu B (2020). Neurologic manifestations of hospitalized patients with coronavirus disease 2019 in Wuhan, China. JAMA Neurol.

[CR95] Marttila RJ, Halonen P, Rinne UK (1977). Influenza virus antibodies in Parkinsonism. Comparison of postencephalic and idiopathic Parkinson patients and matched controls. Arch Neurol.

[CR96] Matías-Guiu J, Gomez-Pinedo U, Montero-Escribano P, Gomez-Iglesias P, Porta-Etessam J, Matias-Guiu JA (2020). Should we expect neurological symptoms in the SARS-CoV-2 epidemic?. Neurologia.

[CR97] McCall S, Henry JM, Reid AH, Taubenberger JK (2001). Infl uenza RNA not detected in archival brain tissues from acute encephalitis lethargica cases or in postencephalitic Parkinson cases. J Neuropathol Exp Neurol.

[CR98] Meisner F, Scheller C, Kneitz S, Sopper S, Neuen-Jacob E, Riederer P, ter Meulen V, Koutsilieri E, German Competence Network HIV/AIDS (2008). Memantine upregulates BDNF and prevents dopamine deficits in SIV-infected macaques: a novel pharmacological action of memantine. Neuropsychopharmacology.

[CR99] Mihara M, Utsugisawa K, Konno S, Tohgi H (2001). Isolated lesions limited to the bilateral substantia nigra on MRI associated with influenza A infection. Eur Neurol.

[CR100] Moein ST, Hashemian SMR, Mansourafshar B, Khorram-Tousi A, Tabarsi P, Doty RL (2020). Smell dysfunction: a biomarker for COVID-19. Int Forum Allergy Rhinol.

[CR101] Moldofsky H, Patcai J (2011). Chronic widespread musculoskeletal pain, fatigue, depression and disordered sleep in chronic post-SARS syndrome; a case-controlled study. BMC Neurol.

[CR102] Murray RS, Brown B, Brian D, Cabirac GF (1992). Detection of coronavirus RNA and antigen in multiple sclerosis brain. Ann Neurol.

[CR103] Nagashima K, Wege H, ter Meulen V (1978). Early and late CNS-effects of corona virus infection in rats. Adv Exp Med Biol.

[CR104] Nagashima K, Wege H, Meyermann R, ter Meulen V (1978). Corona virus induced subacute demyelinating encephalomyelitis in rats: a morphological analysis. Acta Neuropathol..

[CR105] Nagashima K, Wege H, Meyermann R, ter Meulen V (1979). Demyelinating encephalomyelitis induced by a long-term corona virus infection in rats: a preliminary report. Acta Neuropathol.

[CR106] Nataf S (2020). An alteration of the dopamine synthetic pathway is possibly involved in the pathophysiology of COVID-19. J Med Virol.

[CR107] Naughton SX, Raval U, Pasinetti GM (2020). Potential novel role of COVID-19 in Alzheimer's disease and preventative mitigation strategies. J Alzheimers Dis.

[CR108] Netland J, Meyerholz DK, Moore S, Cassell M, Perlman S (2008). Severe acute respiratory syndrome coronavirus infection causes neuronal death in the absence of encephalitis in mice transgenic for human ACE2. J Virol.

[CR109] Nisipeanu P, Paleacu D, Korczyn AD, Watts RL, Koller WC (1997). Infectious and postinfectious parkinsonism. Movement disorders: neurologic principles and practice.

[CR110] Oberländer U, Pletinckx K, Döhler A, Müller N, Lutz MB, Arzberger T, Riederer P, Gerlach M, Koutsilieri E, Scheller C (2011). Neuromelanin is an immune stimulator for dendritic cells in vitro. BMC Neurosci.

[CR112] Oliver KR, Scallan MF, Dyson H, Fazakerley JK (1997). Susceptibility to a neurotropic virus and its changing distribution in the developing brain is a function of CNS maturity. J Neurovirol.

[CR114] Olney JW, Ikonomidou C, Mosinger JL, Frierdich GJ (1989). MK-801 prevents hypobaric-ischemic neuronal degeneration in infant rat brain. Neurosci.

[CR115] Paterson RW, Brown RL, Benjamin L, Nortley R, Wiethoff S, Bharucha T, Jayaseelan DL, Kumar G, Raftopoulos RE, Zambreanu L, Vivekanandam V, Khoo A, Geraldes R, Chinthapalli K, Boyd E, Tuzlali H, Price G, Christofi G, Morrow J, McNamara P, McLoughlin B, Lim ST, Mehta PR, Levee V, Keddie S, Yong W, Trip SA, Foulkes AJM, Hotton G, Miller TD, Everitt AD, Carswell C, Davies NWS, Yoong M, Attwell D, Sreedharan J, Silber E, Schott JM, Chandratheva A, Perry RJ, Simister R, Checkley A, Longley N, Farmer SF, Carletti F, Houlihan C, Thom M, Lunn MP, Spillane J, Howard R, Vincent A, Werring DJ, Hoskote C, Jäger HR, Manji H, Zandi MS, UCL Queen Square National Hospital for Neurology and Neurosurgery COVID-19 Study Group (2020). The emerging spectrum of COVID-19 neurology: clinical, radiological and laboratory findings. Brain.

[CR116] Pinzon RT, Ongko Wijaya V, Buana RB, Al Jody A, Nalla Nunsio P (2020). Neurologic characteristics in coronavirus disease 2019 (COVID-19): a systematic review and meta-analysis. Front Neurol.

[CR117] Poyiadji N, Shahin G, Noujaim D, Stone M, Patel S, Griffith B (2020). COVID-19-associated acute hemorrhagic necrotizing encephalopathy: CT and MRI features. Radiology.

[CR119] Reichard RR, Kashani KB, Boire NA, Constantopoulos E, Guo Y, Lucchinetti CF (2020). Neuropathology of COVID-19: a spectrum of vascular and acute disseminated encephalomyelitis (ADEM)-like pathology. Acta Neuropathol.

[CR120] Roe K (2020). Explanation for COVID-19 infection neurological damage and reactivations. Transbound Emerg Dis.

[CR121] Rogers JP, Chesney E, Oliver D, Pollak TA, McGuire P, Fusar-Poli P, Zandi MS, Lewis G, David AS (2020). Psychiatric and neuropsychiatric presentations associated with severe coronavirus infections: a systematic review and meta-analysis with comparison to the COVID-19 pandemic. Lancet Psychiatry.

[CR122] Rohn TT, Catlin LW (2011). Immunolocalization of influenza A virus and markers of inflammation in the human Parkinson's disease brain. PLoS One.

[CR124] Rossman J (2020) Coronavirus: many patients reporting neurological symptoms. https://www.theconversation.com. Accessed 24 Apr 2020

[CR125] Rothermel M, Carey RM, Puche A, Shipley MT, Wachowiak M (2014). Cholinergic inputs from Basal forebrain add an excitatory bias to odor coding in the olfactory bulb. J Neurosci.

[CR126] Saad M, Omrani AS, Baig K, Bahloul A, Elzein F, Matin MA, Selim MAA, Al Mutairi M, Al Nakhli D, Al Aidaroos AY, Al Sherbeeni N, Al-Khashan HI, Memish ZA, Albarrak AM (2014). Clinical aspects and outcomes of 70 patients with middle east respiratory syndrome coronavirus infection: a single-center experience in Saudi Arabia. Int J Infect Dis.

[CR127] Salmi A, Ziola B, Hovi T, Reunanen M (1982). Antibodies to coronaviruses OC43 and 229E in multiple sclerosis patients. Neurology.

[CR128] Scheller C, Sopper S, Jassoy C, ter Meulen V, Riederer P, Koutsilieri E (2000). Dopamine activates HIV in chronically infected T lymphoblasts. J Neural Transm.

[CR129] Schoser B, Baum P, Boentert M, Dillmann KU, Emmer A, Knauss S, Enax-Krumova E, Grosskreutz J, Güttsches AK, Hellwig K, Holzapfel K, Kornblum C, Lehmann H, Melms A, Meyer T, Petri S, Pilgram L, Reiners K, Saak A, Schäfer J, Schmidt J, Schneider-Gold C, Schons M, Urban PP, Vorgerd M, Young P, Zierz S (2020). SARS-CoV-2/COVID-19 und neuromuskuläre Erkrankungen. DGNeurologie.

[CR130] Schwartz J, Elizan TS (1979). Search for viral particles and virus-specific products in idiopathic Parkinson disease brain material. Ann Neurol.

[CR131] Shea SD, Katz LC, Mooney R (2008). Noradrenergic induction of odor-specific neural habituation and olfactory memories. J Neurosci.

[CR132] Solomon IH, Normandin E, Bhattacharyya S, Mukerji SS, Keller K, Ali AS, Adams G, Hornick JL, Padera RF, Sabeti P (2020). Neuropathological features of Covid-19. N Engl J Med.

[CR133] Sörensen O, Dales S (1985). In vivo and in vitro models of demyelinating disease: JHM virus in the rat central nervous system localized by in situ cDNA hybridization and immunofluorescent microscopy. J Virol.

[CR134] Spinato G, Fabbris C, Polesel J, Cazzador D, Borsetto D, Hopkins C, Boscolo-Rizzo P (2020). Alterations in smell or taste in mildly symptomatic outpatients with SARS-CoV-2 infection. JAMA.

[CR135] Stewart JN, Mounir S, Talbot PJ (1992). Human coronavirus gene expression in the brains of multiple sclerosis patients. Virology.

[CR136] Sungnak W, Huang N, Bécavin C, Berg M, Queen R, Litvinukova M, Talavera-López C, Maatz H, Reichart D, Sampaziotis F, Worlock KB, Yoshida M, Barnes JL, HCA Lung Biological Network (2020). SARS-CoV-2 entry factors are highly expressed in nasal epithelial cells together with innate immune genes. Nat Med.

[CR138] Takahashi M, Yamada T (2001). A possible role of influenza A virus infection for Parkinson's disease. Adv Neurol.

[CR140] van Riel D, Verdijk R, Kuiken T (2015). The olfactory nerve: a shortcut for influenza and other viral diseases into the central nervous system. J Pathol.

[CR141] Varatharaj A, Thomas N, Ellul MA, Davies NWS, Pollak TA, Tenorio EL, Sultan M, Easton A, Breen G, Zandi M, Coles JP, Manji H, Al-Shahi Salman R, Menon DK, Nicholson TR, Benjamin LA, Carson A, Smith C, Turner MR, Solomon T, Kneen R, Pett SL, Galea I, Thomas RH, Michael BD, CoroNerve Study Group Collaborators (2020). Neurological and neuropsychiatric complications of COVID-19 in 153 patients: a UK-wide surveillance study. Lancet Psychiatry.

[CR142] Vetter P, Vu DL, L'Huillier AG, Schibler M, Kaiser L, Jacquerioz F (2020). Clinical features of covid-19. BMJ.

[CR143] von Weyhern CH, Kaufmann I, Neff F, Kremer M (2020). Early evidence of pronounced brain involvement in fatal COVID-19 outcomes. Lancet.

[CR144] Wang D, Hu B, Hu C, Zhu F, Liu X, Zhang J, Wang B, Xiang H, Cheng Z, Xiong Y, Zhao Y, Li Y, Wang X, Peng Z (2020). Clinical characteristics of 138 hospitalized patients with 2019 novel coronavirus-infected pneumonia in Wuhan, China. JAMA.

[CR148] Weiner LP (1973). Pathogenesis of demyelination induced by a mouse hepatitis virus (JHM virus). Arch Neurol.

[CR149] Wichmann D, Sperhake JP, Lütgehetmann M, Steurer S, Edler C, Heinemann A, Heinrich F, Mushumba H, Kniep I, Schröder AS, Burdelski C, de Heer G, Nierhaus A, Frings D, Pfefferle S, Becker H, Bredereke-Wiedling H, de Weerth A, Paschen HR, Sheikhzadeh-Eggers S, Stang A, Schmiedel S, Bokemeyer C, Addo MM, Aepfelbacher M, Püschel K, Kluge S (2020). Autopsy findings and venous thromboembolism in patients with COVID-19. Ann Intern Med.

[CR150] Wu P, Duang F, Luo C, Liu Q, Qu X, Liang L, Wu K (2020). Characteristics of ocular findings of patients with coronavirus disease 2019 (COVID-19) in Hubei Province, China. JAMA Ophthalmol.

[CR151] Xydakis MS, Dehgani-Mobaraki P, Holbrook EH, Geisthoff UW, Bauer C, Hautefort C, Herman P, Manley GT, Lyon DM, Hopkins C (2020). Smell and taste dysfunction in patients with COVID-19. Lancet Infect Dis.

[CR152] Yan CH, Faraji F, Prajapati DP, Boone CE, DeConde AS (2020). Association of chemosensory dysfunction and Covid-19 in patients presenting with influenza-like symptoms. Int Forum Allergy Rhinol.

[CR153] Yang Q, Chen L, He X, Gao Z, Shen X, Bai D (2008). Design and synthesis of cinanserin analogs as severe acute respiratory syndrome coronavirus 3CL protease inhibitors. Chem Pharm Bull (Tokyo).

[CR154] Yang X, Yu Y, Xu J, Shu H, Xia J, Liu H, Wu Y, Zhang L, Yu Z, Fang M, Yu T, Wang Y, Pan S, Zou X, Yuan S, Shang Y (2020). Clinical course and outcomes of critically Ill patients with SARS-CoV-2 pneumonia in Wuhan, China: a single-centered retrospective, observational study. Lancet Respir Med.

[CR155] Yeh EA, Collins A, Cohen ME, Duffner PK, Faden H (2004). Detection of coronavirus in the central nervous system of a child with acute disseminated encephalomyelitis. Pediatrics.

[CR156] Zheng YY, Ma YT, Zhang JY, Xie X (2020). COVID-19 and the cardiovascular system. Nat Rev Cardiol.

[CR157] Zimniak M, Kirschner L, Hilpert H, Seibel J, Bodem J (2020). The serotonin reuptake inhibitor Fluoxetine inhibits SARS-CoV-2. bioRxiv.

